# Estimates of the duration of the early and late stage of gambiense sleeping sickness

**DOI:** 10.1186/1471-2334-8-16

**Published:** 2008-02-08

**Authors:** Francesco Checchi, João AN Filipe, Daniel T Haydon, Daniel Chandramohan, François Chappuis

**Affiliations:** 1 Department of Infectious and Tropical Diseases, London School of Hygiene and Tropical Medicine, Keppel Street, London WC1E7HT, UK; 2 Department of Epidemiology and Population Health, London School of Hygiene and Tropical Medicine, Keppel Street, London WC1E7HT, UK; 3 Division of Environmental and Evolutionary Biology, University of Glasgow, Glasgow G12 8QQ, UK; 4 Médecins Sans Frontières, Swiss section, Rue de Lausanne 78, 1211 Geneva 21, Switzerland; 5 Geneva University Hospitals, Travel and Migration Medicine Unit, Rue Micheli-du-Crest 24, 1211 Geneva 14, Switzerland

## Abstract

**Background:**

The durations of untreated stage 1 (early stage, haemo-lymphatic) and stage 2 (late stage, meningo-encephalitic) human African trypanosomiasis (sleeping sickness) due to *Trypanosoma brucei gambiense *are poorly quantified, but key to predicting the impact of screening on transmission. Here, we outline a method to estimate these parameters.

**Methods:**

We first model the duration of stage 1 through survival analysis of untreated serological suspects detected during Médecins Sans Frontières interventions in Uganda and Sudan. We then deduce the duration of stage 2 based on the stage 1 to stage 2 ratio observed during active case detection in villages within the same sites.

**Results:**

Survival in stage 1 appears to decay exponentially (daily rate = 0.0019; mean stage 1 duration = 526 days [95%CI 357 to 833]), possibly explaining past reports of abnormally long duration. Assuming epidemiological equilibrium, we estimate a similar duration of stage 2 (500 days [95%CI 345 to 769]), for a total of nearly three years in the absence of treatment.

**Conclusion:**

Robust estimates of these basic epidemiological parameters are essential to formulating a quantitative understanding of sleeping sickness dynamics, and will facilitate the evaluation of different possible control strategies.

## Background

Human African trypanosomiasis (sleeping sickness, HAT) has historically been a predominant parasitic infection in Africa, causing many millions of deaths in the late 1800s through early 1900s, and has re-emerged in historical foci after breakdown of control programmes [1]. Two forms of HAT are recognised, due respectively to *Trypanosoma brucei gambiense *(a mainly human disease found mainly in western and central Africa) and *Trypanosoma brucei rhodesiense *(a zoonosis observed in eastern and southern Africa) [2]. Gambiense HAT has an insidious onset and progresses over years, while rhodesiense HAT is fulminant, causing death within a few months of infection [3]. In recent years, 10 000–15 000 HAT cases (mostly gambiense) are reported annually [4], but this is likely an underestimate of the true burden.

Fundamental aspects of the pathogenesis, clinical profile and epidemiology of HAT remain poorly understood. Infecting trypanosomes first colonise the haemo-lymphatic system, evading specific immunity through antigenic variation, but causing only mild and intermittent symptoms – this is known as stage 1 (early or haemo-lymphatic stage). Eventually, parasites cross the blood-brain barrier and cause severe neurological consequences, systemic deterioration and, ultimately, death [5]. The period after blood-brain barrier penetration is called stage 2 (late or meningo-encephalitic stage).

The durations of both disease stages in gambiense HAT, key parameters in the determination of the reproductive ratio of HAT, are not well quantified, and understood only on the basis of informal analysis or anecdote. Both stage 1 and 2 are believed to last from months to years in gambiense disease. However, atypically fast or slow progressions are reported and gambiense HAT, just like rhodesiense, seems to exhibit a wide range of virulence, often following geographical patterns [3,6,7].

In a separate paper (Checchi et al., submitted), we review available evidence on the natural evolution of untreated gambiense HAT, and consider the possible phenomenon of trypano-tolerance, including instances where patients might spontaneously clear their infections or become chronic, asymptomatic carriers. We find that most infections are likely to be pathogenic and fatal if untreated, but that chronic carriage cannot be ruled out based on available evidence, and would, even if occurring at low frequencies, play a crucial role in maintaining transmission even in HAT foci where intensive case detection campaigns are carried out. Trypano-tolerance is a well-described phenomenon in cattle and other animal species [8], and has been postulated by some as an explanation for the mysterious persistence of various historical HAT foci [6,9].

In the present paper, we focus on pathogenic *T.b.gambiense *cases only, which probably constitute the majority of infections, and estimate indirectly the duration of both stage 1 and stage 2 from time of infection to death, in the absence of treatment, based on observations of untreated serological suspects, and the prevalence of the two stages in defined populations. Rigorous and robust estimation of these parameters is crucial to enable quantitative evaluation of the potential of interventions to reduce transmission by minimising the period of human infectiousness to flies [10]. Estimates for *T.b.rhodesiense *have been derived elsewhere [11,12] by analysing patient-reported time from symptom onset to stage 2 presentation or death.

## Methods

### Overall study design

**Figure 1:**
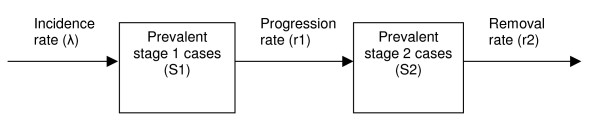
Simple model for the progression of untreated HAT.

The progression of HAT infection from stage 1 to stage 2 and from stage 2 to death or any other outcome, in the absence of any treatment, can be modelled simply (Figure 1). Provided that model parameters specifying the progression rates from stage 1 (r_1_) and stage 2 (r_2_) can be estimated, the mean and median durations of both stages, and their confidence intervals, can easily be calculated. Furthermore, if the numbers of stage 1 and stage 2 cases are at steady state (equilibrium), the ratio of stage 1 to stage 2 prevalence (S_1_/S_2_) will also be constant over time. Assuming equilibrium, S_1_/S_2 _must be directly proportional to the relative durations of stage 1 and stage 2; thus, if one of the two durations is known, the other can be deduced.

The challenge is to identify data from actual HAT foci that can inform model parameters; and in particular, to estimate r_1 _and/or r_2 _whilst maintaining the ethical obligation to treat parasitologically confirmed infections immediately. Here, we use data from Médecins Sans Frontières (MSF) HAT programmes to estimate (i) r_1 _from a survival analysis of serological suspects kept under observation without treatment, and (ii) S_1 _and S_2 _based on the output of active screening sessions, in which communities are tested exhaustively over the space of a few days, yielding the point prevalence of each stage. We then deduce r_2 _from the estimates of r_1_, S_1 _and S_2_.

Analysis was performed with Stata 9.0 software (Stata Corporation, College Station, Texas, USA). Secondary analysis of these operational data was approved by the Ethics Committee of the London School of Hygiene and Tropical Medicine.

### Estimation of stage 1 to stage 2 progression rate (r_1_)

We pooled serological screening and individual patient datasets from six MSF HAT control programmes (Moyo, Uganda, 1986–1993; Adjumani, Uganda, 1991–1996; Arua, Uganda, 1995–2002; Yumbe, Uganda, 2000–2002; Kiri (Kajo Keji), southern Sudan, 2000–2005; Maridi, southern Sudan, 1999–2005). Programmes and datasets have been described previously [13,14]. Briefly, cases are detected through a combination of passive (fixed-centre) and active (mass screening) case detection. Screening relies on the serological Card Agglutination Test for Trypanosomiasis (CATT, Institute of Tropical Medicine, Antwerp, Belgium). Persons positive for the CATT in whole blood go through a complex diagnostic algorithm consisting of a repeat CATT test at progressively higher dilutions, direct microscopy of aspirate from cervical lymph nodes (if present), microscopical examination of blood using one or more concentration techniques (the Quantitative Buffy Coat, Woo, and/or mini-Anion Exchange Column tests), and a lumbar puncture for staging purposes (microscopic evidence of trypanosomes or a white blood cell [WBC] count >5/μL in cerebro-spinal fluid [CSF] leads to stage 2 classification).

Frequently, individuals display a strong CATT reaction but no parasitological evidence of HAT infection and are thus classified as serological suspects. Because the CATT alone is poorly predictive (specificity is around 95% but infection prevalence is usually <5%, giving positive predictive values <50%) [15], treatment is withheld in such cases. Serological suspects are asked to return for repeat testing after three, six, nine, and twelve months, and are treated immediately if found to have parasitologically confirmed HAT, or released from further follow-up if the strong CATT reaction disappears.

Our analysis focussed on stage 1 serological suspects, defined as patients positive at a CATT dilution ≥1:4, but with no parasitological evidence of HAT in any fluids, and a CSF WBC count ≤5/μL. Among patients who were seen at least once after initial screening, possible endpoints at the last follow-up visit were defined as (i) non-case if the CATT became negative or positive at a dilution <1:4, in the absence of other parasitological evidence of HAT, (ii) persistent suspect if positivity at CATT dilution ≥1:4 remained but other tests were still negative, (iii) confirmed stage 1 if blood or gland aspirate became positive and CSF remained negative, or (iv) confirmed stage 2 if parasites were observed in the CSF, if parasites were observed only in blood or lymph fluid but CSF WBC count exceeded 5/μL, or if the CSF WBC count exceeded 20/μL in the presence of a CATT dilution ≥1:4, irrespective of other tests. Only patients who developed either stage 1 or 2 HAT during follow-up (endpoints (iii) and (iv) above) were retained in this study for further survival analysis. Among these, patients reaching endpoint (iv) were considered to have progressed to stage 2, and those with endpoint (iii) to have remained in stage 1 for the duration of follow-up.

Maximum-likelihood estimation was used to fit parametric models to the observed survival data. While estimating confidence intervals for model parameters, possible clustering of survival probabilities within project sites was adjusted for by considering each site as a cluster and taking into account within-cluster correlation of observations. So as to select an optimal model, different hypotheses about the most closely fitting underlying distribution (exponential, Weibull, log-logistic, gamma, and Gompertz) were evaluated using Wald tests if models were nested (for example, the exponential distribution is a particular case of the Weibull), or the Akaike Information Criterion if they were not. Ordinarily, survival analysis assumes that events occur when they are observed (right-censoring). In our case, because follow-up visits took place months apart, we decided against such an assumption, and instead treated observations of stage 2 progression as interval-censored, i.e. considered simply that progression had occurred within a time interval defined by the visit when stage 2 was confirmed and the previous visit (when it was known that the patient had not yet progressed). This method implies likelihood expressions that are somewhat different from the right censoring case [16] and a user-defined Stata module for interval-censored survival analysis was applied [17]. So as to provide some graphical assessment of the goodness of fit of the parametric models, we compared fits of these models to a non-parametric Kaplan-Meier curve of the survival data, with Greenwood 95% confidence intervals, censoring each progression event at the exact mid-point of the interval between the two visits between which it was known to have occurred (a close approximation to interval-censoring).

**Figure 2:**
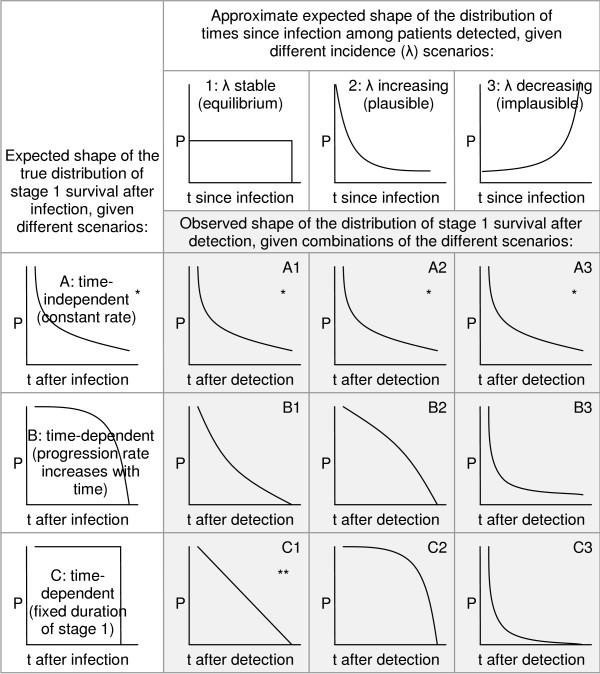
**Expected shapes of the observed distribution of survival times after detection**. Shaded cells A1-C3 show the expected distribution of survival times after detection, given different scenarios for the true distribution of stage 1 survival (A, B and C) and incidence in the pre-detection period (1, 2 and 3). P = proportion of cases remaining in stage 1; t = time; * = exponential function $P(t)=e^{\left(-r_{1}t\right)}$, with mean stage 1 duration = 1/r_1; _** = linear function *P*(*t*) = -*r*_1_*t *with fixed stage 1 duration = 1/r_1_.

The resulting model enables estimation of survival time in stage 1 after detection. However, upon detection patients will have already been infected for some time; the distribution of these times since infection is unknown, and is itself dependent on incidence trends in the pre-detection period. If incidence is stable, this distribution will be uniform; if incidence is increasing, proportionately more will have been infected recently. As illustrated in Figure 2, if survival is dependent on time since infection, the observed shape of the distribution of survival times after detection will reflect both the distribution of times since infection (itself a result of past incidence trends) and the true distribution of survival in stage 1 (i.e. r_1_), as in scenarios B1, B2, B3, C1, C2 and C3. If survival is in reality time-independent (constant rate), both the true and observed distributions will be exponential (scenarios A1, A2 and A3), and will share the same parameter value, irrespective of whether incidence is stable or not.

### Estimation of S_1 _and S_2_

Data on active screening sessions were available from the Adjumani, Arua, and Kiri programmes, and were used to provide realistic values for S_1 _and S_2 _in situations unmodified by control. Screening sessions were retained for analysis only if they were the first to be conducted in the respective villages, reached a coverage (persons screened/total population) ≥70% (a commonly used target in HAT programmes), and yielded at least one case of HAT. The mean and median S_1 _and S_2_, and their ratio, were calculated after weighting for village population size. We also did sensitivity analysis to see whether passive case detection in the pre-screening period affected the S_1 _to S_2 _ratio.

### Estimation of stage 2 removal rate (r_2_)

Assuming that no treatment is available, the prevalence of cases in stage 2 at time t is given by the prevalence at the previous time point (t - δt), plus new progressions from stage 1, minus stage 2 patients removed, such that:

*S*_2_(*t*) = *S*_2_(*t *- *δt*) + *S*_1_(*t *- *δt*)*r*_1 _- *S*_2_(*t *- *δt*)*r*_2_

Assuming equilibrium conditions, *S*_2_(*t*) = *S*_2_(*t *- *δt*) = *S*_2 _and *S*_1_(*t*) = *S*_1_(*t *- *δt*) = *S*_1_. Thus, solving for r_2 _gives

$$r_{2}=r_{1}\left(\frac{S_{1}}{S_{2}}\right)$$

In an epidemiological scenario meeting the above assumptions, the S_1_/S_2 _ratio is thus linearly proportional to the relative duration of r_2 _and r_1_.

## Results

### Description of included and excluded patient cohorts

Out of 7742 patients listed as suspects in the six databases, 1079 were not eligible for the analysis: 385 (5.0%) had laboratory findings compatible with confirmed stage 1 or 2 HAT and were in fact treated; 577 (7.5%) were stage 2 serological suspects; and 117 (1.5%) had an unclear stage due to missing variables. This left 6663 (86.1%) patients meeting stage 1 suspect criteria.

Baseline characteristics (Table 1) differed significantly across sites (p < 0.001 for age; p < 0.001 for proportion with zero WBC in CSF, with high levels in Maridi). Follow-up rates were low everywhere (Table 1) with the exception of Arua. Of the 6663 patients eligible, 2250 (33.8%) attended one follow-up visit, 580 (8.7%) attended two, 232 (3.5%) three, 114 (1.7%) four, and two (0.03%) five visits. Overall, 47.7% of suspects returned for follow-up at least once. Attendance declined with time (however, the low follow-up rates at months 9 and 12 partly reflect the fact that by then many patients had been released from follow-up after becoming CATT negative). Among patients seen once, person-time under observation was roughly comparable across sites.

**Table 1:** Baseline characteristics and rates of follow-up of stage 1 serological suspects, by site

	Adjumani, Uganda	Arua, Uganda	Moyo, Uganda	Yumbe, Uganda	Kiri, s. Sudan	Maridi, s. Sudan	Total
		
Number of patients	1140	2215	296	81	2201	730	6663
		
Baseline characteristics							
Median age (IQR)	20 (12–32)	25 (14–35)	22 (12–36)	25 (13–40)	21 (13–32)	23 (15–35)	20 (12–33)
Number female (%)	595 (52.2)	1148 (51.8)	166 (56.1)	44 (54.3)	1231 (55.9)	386 (52.8)	3570 (53.6)
Number screened actively (%)	853 (74.8)	1362 (61.5)	239 (80.7)	14 (17.3)	926 (42.1)	165 (22.6)	3559 (53.4)
Number with zero WBC in CSF† (%)	607 (53.2)	1027 (46.4)	194 (65.5)	21 (25.9)	1052 (47.8)	9 (0.02)	2910 (43.7)
		
Follow-up rates							
Not seen during follow-up (%)	660 (57.9)	410 (18.5)	249 (84.1)	47 (58.0)	1502 (68.2)	617 (84.5)	3485 (52.3)
Seen at least once (%)	480 (42.1)	1805 (81.5)	47 (15.9)	34 (42.0)	699 (31.8)	113 (15.5)	3178 (47.7)
Seen at 3 months (%)	250 (21.9)	1180 (53.3)	23 (7.8)	25 (30.9)	412 (18.7)	63 (8.6)	1953 (29.3)
Seen at 6 months (%)	112 (9.8)	570 (25.7)	15 (2.1)	11 (13.6)	135 (6.1)	15 (2.0)	850 (12.8)
Seen at 9 months (%)	61 (5.3)	299 (13.5)	2 (0.7)	4 (4.9)	53 (2.4)	11 (1.5)	430 (6.5)
Seen at 12 months (%)	63 (5.5)	366 (16.5)	6 (2.0)	6 (7.4)	60 (2.7)	9 (1.2)	510 (7.6)
Median person-days of observation†† (IQR)	169 (98–277)	177 (97–328)	110 (97–201)	151 (98–271)	128 (96–209)	117 (93–198)	158 (97–281)

IQR = inter-quartile range; WBC = white blood cells; CSF = cerebrospinal fluid

†Note that all patients had ≤5 WBC/μL, as per stage 1 suspect definition. Presence of low WBC densities is generally not considered evidence of CSF infection, and the threshold of 5 cells/μL is also used in neurology.

††Among patients seen at least once.

Marked differences in patient endpoints were evident across sites (Table 2). Most persons lost their strong CATT reactivity during follow-up (non-cases), about a third remained suspects, while parasitologically confirmed HAT was noted in only a minority. Overall, 199 suspects were confirmed as stage 1 during follow-up, and 99 progressed to confirmed stage 2 (Table 2), leaving 298 "true" HAT cases among whom survival analysis was conducted.

**Table 2:** Endpoints during follow-up among stage 1 serological suspects who attended at least one control visit, by site

	Adjumani, Uganda	Arua, Uganda	Moyo, Uganda	Yumbe, Uganda	Kiri, s. Sudan	Maridi, s. Sudan	Total
		
Not included in survival analysis							
Dead (%)	0 (0.0)	0 (0.0)	0 (0.0)	0 (0.0)	1 (0.1)	0 (0.0)	1 (0.03)
Non-case (CATT reactivity waned) (%)	279 (58.1)	1198 (66.4)	13 (27.7)	21 (61.8)	326 (46.6)	51 (45.1)	1888 (59.4)
Persistent suspect (%)	102 (21.3)	491 (27.2)	10 (21.3)	11 (32.4)	330 (47.2)	47 (41.6)	991 (31.2)
		
Included in survival analysis							
Confirmed stage 1 HAT (%)	61 (12.7)	87 (4.8)	18 (38.3)	1 (2.9)	23 (3.3)	9 (8.0)	199 (6.3)
Progressed to stage 2 HAT (%)	38 (7.9)	29 (1.6)	6 (12.8)	1 (2.9)	19 (2.7)	6 (5.3)	99 (3.1)
		
Total	480	1805	47	34	699	113	3178
		
Median number of days between detection as stage 1 serological suspect and stage 2 diagnosis (IQR)	179 (99–319)	206 (181–409)	213 (103–417)	198 (single observation)	135 (109–221)	64 (28–168)	189 (104–319)

The 298 patients retained differed systematically from the remaining 6365 stage 1 suspects who were not confirmed as stage 1 or 2 during follow-up, and thus excluded: they were slightly younger (mean age 23.4 years versus 25.0 years, p = 0.029), a lower proportion had a WBC count of zero in their CSF (109 [36.6%] versus 2801 [44.0%], p = 0.011), and a lower proportion were screened actively (132 [44.3%] versus 3427 [53.8%], p = 0.001). Patients followed up at least once also differed systematically from those never seen again: more were female (1528/3178 or 48.1% versus 1565/3485 or 44.9%; p = 0.01); more had a zero-WBC count in CSF (1438/3178 or 45.2% versus 1472/3485 or 42.2%; p = 0.01); and considerably more were positive at a CATT dilution >1:4 (2165/2713 or 79.8% versus 1468/2305 or 63.7%; p < 0.001).

### Stage 1 duration

**Figure 3:**
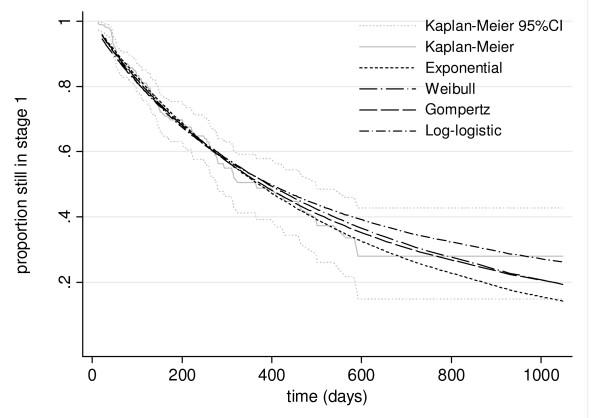
**Fitted interval-censored stage 1 survival models**. For comparison purposes, the figure also superimposes a Kaplan-Meier survival curve (with Greenwood 95% confidence intervals) based on censoring progression events at the mid-point between the two visits between which the event is known to have occurred.

After maximum likelihood estimation, a simple exponential model fit the data no worse (p > 0.20 for all comparisons among nested models) than any more complex alternatives (Weibull, log-logistic, Gamma, and Gompertz), as evidenced visually (Figure 3), and was thus retained. Because distribution of survival times post detection is exponential, the overall function for survival in stage 1 must also be exponential and have the same parameter (Figure 2). The model thus yields the following function for the proportion P of cases remaining in stage 1 up to time t:

$$P(t)=e^{\left(-r_{1}t\right)}$$

where r_1_, the daily rate of progression to stage 2, is 0.0019 (95%CI 0.0012 to 0.0028). Accordingly, the mean duration of stage 1 is 1/r_1_, or 526 days (95%CI 357 to 833), corresponding to about one and a half years. The median duration is ln(2)/r_1_, or 364 days (95%CI 248 to 578).

The estimate of r_1 _is higher for patients detected though passive screening at the HAT centre (0.0021, 95%CI 0.0012 to 0.0038) than for patients detected actively in the community (0.0016, 95%CI 0.0013 to 0.0020). However, the difference is non-significant.

### Stage 2 duration

Overall, 88 active screening sessions were eligible for analysis (Table 3). Infection prevalences were comparable in Adjumani and Kiri, but considerably lower in Arua. Nevertheless, the ratio of stage 1 and 2 prevalence was similar across sites, and the overall average was close to unity (1.04) considering the ratio of means, while a ratio of medians yielded 1.25.

**Table 3:** Details of eligible active screening sessions and stage 1 and 2 prevalences

	Adjumani	Arua	Kiri	Total
				
Eligible screening sessions (n)	28	35	25	88
Years of intervention	1992–1994	1997–2000	2000–2004	-
Total population screened	17 550	78 268	16 231	112 049
Village population: median (IQR)	700 (417–1418)	2209 (1777–2558)	199 (115–260)	861 (287–2148)
				
Pre-screening passive detection rate: cases per 1000 person-months, weighted median (range)	0.32 (0.00–6.33)	0.07 (0.00–2.24)	0.78 (0.00–30.46)	0.12 (0.00–30.46)
				
Stage 1 prevalence (S_1_): weighted mean % (range, median)	0.68 (0.00–5.67, 0.28)	0.14 (0.00–0.84, 0.07)	0.54 (0.00–2.31, 0.19)	0.24 (0.00–5.67, 0.10)
Stage 2 prevalence (S_2_): weighted mean % (range, median)	0.66 (0.00–6.67, 0.20)	0.12 (0.00–1.02, 0.07)	0.62 (0.00–6.48, 0.25)	0.23 (0.00–6.67, 0.08)
S_1 _to S_2 _ratio	1.03	1.17	0.87	1.04

Using Equation 2, the stage 2 removal rate r_2 _was thus very close to r_1_: 0.0020 per day (95%CI 0.0013 to 0.0029). Accordingly, the mean duration of stage 2 is 1/0.0020 or 500 days (95%CI 345 to 769), and the mean total duration of stage 1 and 2 combined is 1026 days (95%CI 702 to 1602), or approximately 2 years and 10 months. Similarly, the median stage 2 duration is 347 days (95%CI 239 to 533), giving a total median gambiense HAT duration of 711 days (95%CI 487 to 1111), or almost two years.

Sensitivity analysis to compare S_1_/S_2 _ratios according to the pre-screening passive detection rate did not show significant differences (p = 0.41 for detection rate <1 vs ≥1 per 1000 person-months, Kruskal-Wallis test).

## Discussion

Our estimate of the "natural" duration of gambiense HAT (almost three years, evenly split between stage 1 and 2) helps to refine commonly held views that both stages last 'months to years', and is broadly consistent with ranges provided by experts: "several months to two years" for stage 1 [7], and, for stage 2, "four months to one year" [7] or "from one to three years" [3]. Fèvre et al. did survival analysis on 96 gambiense patients reported in the historical literature, and estimated a median duration of three years [18].

To our knowledge this is the first attempt to quantify the duration of stage 1 and 2 specifically. Furthermore, the distribution of stage 1 survival post detection has a clear exponential shape. As shown in Figure 2, this observation is most consistent with a constant, i.e. time-independent rate of progression, irrespective of incidence trend before detection (scenarios A1, A2 and A3), rendering our estimates entirely robust in this respect. Distributions approximating an exponential could also arise if incidence is stable, and the progression rate increases with time (scenario B1), or if incidence is decreasing (B3 or C3). However, the latter is implausible: in all sites, MSF intervened because incidence was credibly reported to be increasing. Our findings are least consistent with a time-dependent progression rate in a scenario of increasing incidence, which would yield strongly convex distributions (B2 and C2).

The mathematical property of constant r_1 _is crucial to interpreting observed variation in the virulence of HAT: exponential distributions allow for a tail of unusually long durations, reports of which abound in the literature (Checchi et al., submitted). For example, using our estimated survival function, 3% of cases would be expected to remain in stage 1 for >5 years. Such cases should probably no longer be considered extraordinary, but simply a result of the intrinsic pattern of disease variability. The implication for control is that a small but important proportion of cases can carry the infection for many years, and, if undetected by active screening rounds, could maintain a tiny but persistent infectious reservoir even when local elimination appears all but achieved. Mop-up active screening rounds, even years after transmission in a HAT focus is considered to have been brought under control, could thus play a vital role in reducing the risk of epidemic resurgence, something passive case detection might by itself never achieve.

If stage 2 invariably progresses to death, as is widely assumed, then r_2 _is the specific HAT death rate after stage 2 initiation, and 1/r_2 _is the life expectancy of stage 2. To our knowledge only one other study has attempted to estimate this parameter: Jusot et al. re-analysed observations by Greggio on the survival of absconded HAT patients diagnosed at a Belgian Congo hospital between 1907 and 1915 [19]. They showed an exponential decay of survival, and estimated a daily death rate of 0.00235 (mean duration = 425 days), very close to our estimate.

### Potential Biases

**Figure 4:**
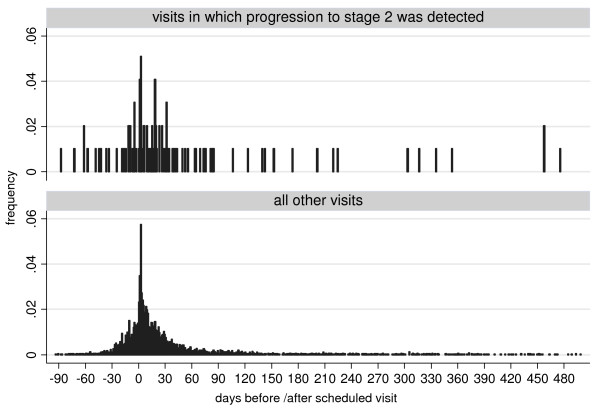
**Timing of actual follow-up visits with respect to the scheduled date**. Data are provided for visits at which progression to stage 2 was detected (n = 99), as well as all other visits (n = 4774).

Our findings are subject to six main potential sources of bias, which mainly affect the estimates of stage duration:

1. A systematic delay in ascertainment of stage 2 progression, leading to an underestimation of stage 1 duration, could have occurred if patients had presented to the treatment centre only when CNS involvement had become symptomatic. Interval censoring removes part of this potential bias by considering only the interval during which the event occurred. Furthermore, only 29.3% (1458/4971) of follow-up visits in our entire cohort of 6663 patients occurred >30 days earlier or later than scheduled, suggesting that event ascertainment was mainly driven by the follow-up schedule, not symptom onset. There was a mean delay of 34 days between the scheduled date of follow-up and the date when the visit actually took place. This delay was greater when patients were found to have progressed to stage 2 at the visit (56 days versus 34 days for all other visits; p = 001 for difference of means; p = 0.070 for difference of medians), but this is a small difference considering HAT evolves over many months. The distribution of visit timing with respect to the scheduled date was similar whether or not progression to stage 2 was detected at the visit (Figure 4).

2. About half of analysis-eligible patients were followed up, each for an average of five months. At baseline, this group had a higher prevalence of strong CATT reactivity than defaulters, suggesting it harboured more true HAT cases. This difference should have been eliminated by virtue of including only confirmed stage 1 or 2 patients in the survival analysis. Stage 1 duration may nonetheless have been underestimated if patients progressing to stage 2 and thus feeling ill were more likely to present for follow-up; however, this pattern may be unlikely given that progression events occurred around scheduled visits. It is also possible that patients progressing may have stayed away from MSF HAT centres due to a perception that the programme had failed to treat them: in this case, duration would be overestimated.

3. Our analysis assumes that suspects have the same rate of progression to stage 2 as 'typical' confirmed stage 1 patients. In fact, CATT serological suspects are a complex population [20], probably composed of a mixture of false parasitological negatives and false serological positives. False negatives would be due to (i) testing during the pre-patent phase (first 1–2 weeks after the tsetse bite); (ii) low-virulence cases featuring a parasite density below the detection threshold of microscopy; (iii) testing when parasite density is at its minimum, as part of its well-described undulating pattern, and thus below the detection threshold; or (iv) laboratory error. False positives would result from (i) CATT cross-reaction with other trypanosome species (some of which are known to cause transient and possibly even pathogenic infections), other parasites such as leishmania and *Plasmodium spp*., or rheumatoid factors [21]; (ii) past HAT infection (antibodies can survive for up to 3 years) [22]; or (iii) error in CATT performance. So as to remove as many of the false positives as possible and isolate a population most likely to comprise typical pathogenic infections, we restricted the denominator at risk to suspect cases that were indeed true HAT cases, as evidenced by stage 1 or 2 parasitological confirmation during follow-up. Arguably, we could have retained in the analysis individuals who remained suspects until the end of follow-up, and the survival function would have been markedly different, yielding a far longer stage 1 duration (r_1 _= 0.00014, or about 20 years), which does not seem plausible. However, the vast majority of these cases have been shown in other studies to be false positives by both parasitological and PCR techniques [20,23-25]. Were suspects progressing more slowly than other cases, the true stage 1 duration would be shorter than our estimate: however, any difference is unlikely to be great, since a much faster r_1 _than the one we measured would yield durations of only a few months, consistent with empirical observations of rhodesiense, but not gambiense HAT, for which one to three years are the norm (Checchi et al, submitted).

4. Our estimate of r_2 _should be regarded as less robust and potentially more biased than that of r_1_, since it relies on an assumption of stable incidence, which is atypical in HAT (indeed, past models of HAT transmission have suggested unstable incidence and lack of equilibrium might be an inherent feature of HAT dynamics) [19,26,27]. A rising incidence, which is more plausible, would skew the S_1_/S_2 _ratio upwards, and result in an overestimation of r_2_, i.e. underestimated stage 2 duration. This bias is probably not large, since all foci in this analysis featured relatively low prevalence (<2–3%), suggesting incidence may have been rising slowly. However, our deduced stage 2 duration should be probably regarded as a lower-end estimate.

5. So as to assemble a sufficiently large cohort of patients, we combined datasets from various HAT foci. We assumed that this cohort would be representative of the population of HAT patients in these foci. However, HAT foci might differ in several ways. Particularly, incidence trends might have been divergent (e.g. increasing in one site and decreasing in another), affecting the stable incidence assumption differently; and the profile of serological suspects, including the prevalence of true HAT stage 1 cases (see point 3 above) might vary due to heterogeneity in incidence, coverage and timeliness of case detection or diagnostic sensitivity across sites: unequal representation of the different foci in the overall cohort, due merely to the duration and coverage of the respective treatment programmes and to incidence during the programme durations, would bias results towards the patterns in foci that are over-represented in the cohort.

6. Estimates here apply to strains of *T. gambiense *present in Sudan and Uganda. More or less virulent strains encountered elsewhere might feature different progression and removal rates.

## Conclusion

Our quantitative understanding of the dynamics of HAT is poor relative to that of many other infectious diseases. This is partly a consequence of imprecise knowledge of the fundamental parameters that govern HAT disease dynamics. Here we have estimated two of these key parameters based on substantial datasets, providing estimates of the true duration of untreated infections. These parameter estimates are essential pre-requisites to the development of formal approaches for the quantitative evaluation of different strategies to control this neglected disease. The duration of infection and thus infectiousness is a fundamental determinant of HAT's reproductive ratio, and present control strategies essentially work by reducing r_1 _and r_2_: mathematical models will only offer reliable predictions of the impact of different screening strategies if they incorporate realistic values for r_1 _and r_2 _[19]. Furthermore, the exponential property of stage 1 to stage 2 progression determines the distribution of asymptomatic infection durations, and therefore the probability that some long-duration cases will fall through the net of case detection before they become symptomatic, and seed renewed HAT outbreaks once control is relaxed. These quantitative dynamic processes should inform any future policies to control and possibly eliminate gambiense HAT.

## Abbreviations

CATT, Card Agglutination Test for Trypanosomiasis; CNS, Central nervous system; CSF, Cerebrospinal fluid; HAT, Human African trypanosomiasis; IQR, Inter-quartile range; MSF, Médecins Sans Frontières; PCR, Polymerase Chain Reaction; S_1_, point prevalence of HAT cases in stage 1 of the disease; S_2_, point prevalence of HAT cases in stage 2 of the disease; λ, incidence rate; r_1_, rate of progression from stage 1 to stage 2; r_2_, rate of removal from stage 2 (through death or spontaneous cure); P(t), proportion of HAT cases still in stage 1 at time t after infection; t, time; WBC, white blood cells.

## Competing interests

The author(s) declare that they have no competing interests.

## Authors' contributions

F Checchi designed the study, carried out analyses and wrote this paper. J Filipe, D Haydon and D Chandramohan assisted in data analysis and interpretation. F Chappuis was involved in study design and interpretation. All authors made substantial contributions to the manuscript.

## Pre-publication history

The pre-publication history for this paper can be accessed here:


